# Organ-, inflammation- and cancer specific transcriptional fingerprints of pancreatic and hepatic stellate cells

**DOI:** 10.1186/1476-4598-9-88

**Published:** 2010-04-23

**Authors:** Mert Erkan, Nadine Weis, Zheng Pan, Christian Schwager, Tamar Samkharadze, Xiaohua Jiang, Ute Wirkner, Nathalia A Giese, Wilhelm Ansorge, Jürgen Debus, Peter E Huber, Helmut Friess, Amir Abdollahi, Jörg Kleeff

**Affiliations:** 1 Department of General Surgery, Technische Universität München, Munich, Germany; 2 Department of Radiation Oncology, German Cancer Research Center (DKFZ) and University of Heidelberg, Heidelberg, Germany; 3 Department of General Surgery, University of Heidelberg, Heidelberg, Germany; 4 Center of Cancer Systems Biology, Dept. of Medicine, Caritas St. Elizabeth's Medical Center, Tufts University School of Medicine, Boston, Massachusetts 02135-2997, USA; 5 Children's Hospital Boston, Vascular Biology Program & Harvard Medical School, Department of Surgery, Karp Family Research Laboratories, Boston, Massachusetts 02115, USA

## Abstract

**Background:**

Tissue fibrosis is an integral component of chronic inflammatory (liver and pancreas) diseases and pancreatic cancer. Activated pancreatic- (PSC) and hepatic- (HSC) stellate cells play a key role in fibrogenesis. To identify organ- and disease-specific stellate cell transcriptional fingerprints, we employed genome-wide transcriptional analysis of primary human PSC and HSC isolated from patients with chronic inflammation or cancer.

**Methods:**

Stellate cells were isolated from patients with pancreatic ductal adenocarcinoma (n = 5), chronic pancreatitis (n = 6), liver cirrhosis (n = 5) and liver metastasis of pancreatic ductal adenocarcinoma (n = 6). Genome-wide transcriptional profiles of stellate cells were generated using our 51K human cDNA microarray platform. The identified organ- and disease specific genes were validated by quantitative RT-PCR, immunoblot, ELISA, immunocytochemistry and immunohistochemistry.

**Results:**

Expression profiling identified 160 organ- and 89 disease- specific stellate cell transcripts. Collagen type 11a1 (*COL11A1*) was discovered as a novel PSC specific marker with up to 65-fold higher expression levels in PSC compared to HSC (p < 0.0001). Likewise, the expression of the cytokine *CCL2 *and the cell adhesion molecule *VCAM1 *were confined to HSC. *PBX1 *expression levels tend to be increased in inflammatory- vs. tumor- stellate cells. Intriguingly, tyrosine kinase *JAK2 *and a member of cell contact-mediated communication *CELSR3 *were found to be selectively up-regulated in tumor stellate cells.

**Conclusions:**

We identified and validated HSC and PSC specific markers. Moreover, novel target genes of tumor- and inflammation associated stellate cells were discovered. Our data may be instrumental in developing new tailored organ- or disease-specific targeted therapies and stellate cell biomarkers.

## Introduction

Emerging body of data suggest a critical role for stellate cells in the pathophysiology of pancreatic cancer and chronic inflammatory diseases [1-5]. Hepatic stellate cells (HSC) were first described by Karl von Kupffer in 1876, however similar cells in the pancreas were first observed in 1980s [1,3,6]. In 1998 Bachem and Apte isolated and cultured pancreatic stellate cells (PSC) [7,8]. Morphologic, functional and gene expression studies revealed that PSC resemble HSC characteristics and therefore may possibly share a common origin [3,9]. However, the origin of stellate cells is still controversially debated. Mesenchymal [10,11], endodermal [12,13] as well as neuroectodermal origins [14-16] are suggested. Further, it is postulated that in the diseased organ, stellate cells are transformed from their quiescent precursors, or recruited from local fibroblasts, bone marrow derived cells or generated *via *epithelial-mesenchymal transformation [1,3,17].

HSC represent 5-8% of all human liver cells and reside in the space of Disse [1]. In contrast to quiescent HSC, activated HSC lack cytoplasmic lipid droplets containing retinyl esters and long cytoplasmic processes. Their activation or trans-differentiation is regulated by paracrine and autocrine loops of growth factors which are associated with pathological conditions such as liver injury, cirrhosis and cancer [1,2]. Stellate cell over-activity can severely impair organ function due to excessive contraction and abundant extracellular matrix protein deposition. Moreover, it is becoming clearer that myofibroblasts found in the activated stroma of epithelial tumors significantly impact tumor behavior [5,18]. Tumor-stroma interactions influence both the progression of cancer and tumor responses to cancer therapies [4,5,18-21]. Since conventional therapies are far from cure, new targeted therapies appear as promising alternatives or adjuncts [22]. Indeed, the tumor microenvironment and the desmoplastic reaction observed in pancreatic ductal adenocarcinoma (PDAC) have attracted enormous scientific attention and emerged as a critical therapeutic target [19,23,24].

To selectively and specifically target HSC or PSC in chronic inflammatory diseases or in cancer, a better molecular characterization of these cells is required. In an attempt to identify organ- and disease-specific transcripts, we isolated stellate cells from a total of 22 patients with primary PDAC, chronic pancreatitis, liver cirrhosis, and liver metastasis of PDAC. Genome-wide transcriptional analysis was employed and novel candidate tumor-, inflammation- or organ-specific stellate cell genes were identified and validated in the tissues of these patients by real time quantitative RT-PCR, immunohistochemistry, immunocytochemistry, ELISA and immunoblot analyses.

## Materials and methods

### Pancreatic tissues and human pancreatic stellate cell cultivation

The use of human material for the analysis was approved by the local ethics committee of the University of Heidelberg, Germany, and written informed consent was obtained from all patients. Sterile tissues were obtained immediately after surgical resection from five patients with PDAC, six patients with chronic pancreatitis (CP), five patients with liver cirrhosis (LC) that underwent liver transplantation, and six patients with liver metastasis (LM) of pancreatic cancer. During tissue collection, freshly removed samples were either snap frozen in liquid nitrogen and stored at -80°C for protein and DNA extraction or preserved in RNA-later solution for future RNA extraction. A portion of the samples was also fixed in 4% parafomaldehyde solution for 12-24 h and then embedded in paraffin for histological analysis. Human stellate cell isolation and cultivation were performed under sterile conditions for all cell types by using the outgrowth method as described initially by Bachem et al. [8]. Briefly; passage-1 was described as the first lot of cells growing out from fibrotic blocks of pancreatic tissues seeded in 10 cm Petri dishes. To prevent bias, the number of blocks was kept constant (30 blocks with 2-3 mm diameter/per 75 cm^2^). Passage-2 is a 1:2 division of these cells into two new T75 cm^2 ^flasks. When passage-2 cells reached confluency, they were aliquoted and frozen. All cells used were passage-3 after thawing a clone of frozen passage-2. Purity of stellate cells was routinely checked by immunocytochemistry and immunofluorescence analyses (Additional file1). All passages used were controlled and no morphologically different subpopulation was detected.

### Total RNA isolation

To prevent passage dependent variations, third passages of PSC and HSC were used for all analyses. Total RNA from 80% confluent stellate cells in 10 cm Petri dishes was isolated by organic extraction with the phenolic Trizol reagent as described [25]. The Agilent 2100 Bioanalyzer (Agilent Technologies Inc. Santa Clara, CA) was used for the quality control of the isolated total RNA and amplified RNA (aRNA) by capillary electrophoretic separation [26].

### Genome-wide expression profiling

Genome-wide expression profiling was done using 51K Human Unigene III cDNA microarrays. The microarrays were designed, generated, and hybridized as described previously [24,26,27]. Each sample was hybridized against Human Universal Reference Total RNA (# 636538, BD Clontech, Heidelberg, Germany). Expression profiling was performed as previously described with minor modifications [24,26,27]. Linear amplification from 2 μg total RNA was performed using the "MessageAmp II aRNA Amplification Kit" (Ambion, #1751). From amplified RNA, 5 μg were used for indirect labeling by incorporation of aminoallyl modified nucleotides and chemical attachment of free reactive fluorescent Cy3- or Cy5-dye (Atlas Glass fluorescent labelling kit, BD Biosiences Catalogue #K1037-1; Cy5-dye, Cy3-dye, GE Healthcare UK Limited, #Q15108, #Q13108). Corresponding Cy3- and Cy5- labeled probes and competitor DNA (5 μg human Cot-DNA, Invitrogen; 5 μg poly-dA, Amersham) were combined, diluted in hybridization buffer to a final volume of 80 μl (50% Formamide, 6× SSC, 0.5% SDS, 5× Denhards), and denatured for 5 min at 95°c prior to hybridization. Prehybridization was performed at 42°C for 20 min in 6× SSC, 0.5% SDS, 1% BSA. Slides were rinsed in H_2_O and spotted probes were denatured by incubating the slide for 2 min in 90°C H_2_O. Hybridization probe was added and static hybridization performed at 42°C for 16 h. Excess of probe was removed by washing in 2 × SSC, 0.5%SDS at 42°C for 5 min, then in 0.2 × SSC, 0.5%SDS at 42°C for 15 min and finally in isopropanol for 30s at RT. Slides were scanned with Agilent Microarray Scanner and image processing was done using the "Chipskipper" software. Data were stored in MO-MEX database Bloader that enables direct submission of large batches of MIAME complaint expression profiling data to the ArrayExpress database. Microarray data are available online at ArrayExpress http://www.ebi.ac.uk/arrayexpress under the accession no. E-TABM-625.

### Statistical analysis of the microarray data

Generation of expression matrices, data annotation, filtering, and processing were done using TableButler software package http://www.oncoexpress.de/software/tablebutler. All microarray statistics including *t-test *with permutation analysis, Pavlidis template matching (PTM), and cluster analysis were done using the Java based software package TIGR MultiExperiment Viewer (TMEV) version 3.01 http://www.tm4.org/mev.html. Genes were defined as differentially expressed between two sets of samples if a two-class permutation based *t-test *(number of permutations = 1000) yielded a p-value of less than 0.05 after standard Bonferroni correction for multiple hypothesis testing. Subsequently, regulated genes were sorted by hierarchical clustering (HCL). PTM allows the specification of a template expression profile for a gene by designating relative gene expression ratios for each condition. The data sets are searched for matches to this template, i.e. for the specific expression pattern. Gene expression data were filtered for genes that matched each template based on a Pearson correlation coefficient r ≥ 0.70. The obtained gene expression profiles were visualized as heatmaps.

### Real time quantitative PCR

All reagents and equipment for mRNA/cDNA preparation were purchased from Roche Applied Science Diagnostics (Mannheim, Germany). mRNA extractions were prepared by automated isolation using the MagNA Pure LC instrument and isolation kit I. cDNA was prepared using the first-strand cDNA synthesis kit (AMV) according to the manufacturer's instructions. Real-time PCR was performed with the Light Cycler Fast Start DNA SYBR Green kit. All primers were obtained from Search-LC (Heidelberg, Germany). The calculated number of specific transcripts was normalized to 10,000 copies of the housekeeping gene PPIB (*peptidylprolyl isomerase B*, also known as *cyclophilin B*), and expressed as number of copies per μl of input cDNA. Statistical comparisons were made by Mann-Whitney U test. A P-value less than 5% was regarded as significant.

### Immunohistochemistry and immunocytochemistry analyses

IHC was performed as described before [28]. Antibodies used and the dilution ratios are shown in Table 1. Immunocytochemistry analysis was performed using a previously described protocol [29].

**Table 1:** The antibodies used and the appropriate dilutions.

	COL11a1	VCAM1	CELSR3	Pbx1
**Company**	Santa Cruz (sc-68853)	Santa Cruz (sc-8304)	Abcam (ab12958) Santa Cruz (sc-46849)	Abcam (ab12001)

**Host**	Rabbit	Rabbit	Rabbit (Abcam) Goat (Santa Cruz)	Rabbit

**Immunohistochemistry**	1:500	1:200	1:100 (Abcam)	1:100

**Immunoblot**	na	1:250	1:250 (Santa Cruz)	1:100

**Immunocytochemistry**	1:100	Na	Na	na

na: not applicable

### Immunoblot analysis and densitometry

To keep passage dependent variations at minimum, third passages of PSC and HSC were used for analysis. Stellate cells were grown in Petri dishes until 80% confluency under standard conditions. Protein extraction was carried out as published before [28]. Immunoblot analyses and the densitometric analysis of the blots were done as described previously [30]. Antibodies used and the appropriate dilution are shown in Table 1. Graphic results are expressed after normalization to the housekeeping gene GAPDH as percent change of the appropriate control.

### ELISA

Cells were grown in 10-cm plates until 80% confluence. Serum free medium was kept on the cells for 48 h. Supernatants and total cell lysates were extracted and frozen at -80°C until use. For the quantification of CCL2, a commercially available ELISA kit (Human MCP-1 ELISA, 555179, BD Biosciences, Heidelberg, Germany) was used according to the manufacturers' recommendations.

## Results

### Evaluation of gene expression profiles

**Figure 1:**
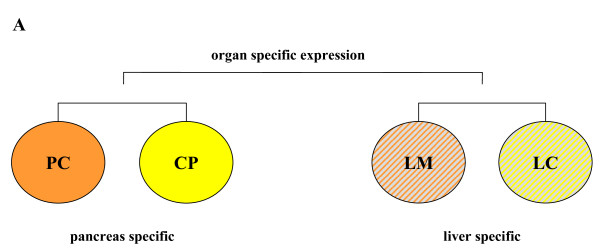
**Schematic expression of the hypothesis: Organ specific expression profile (pancreas vs. liver) of stellate cells**.

**Figure 2:**
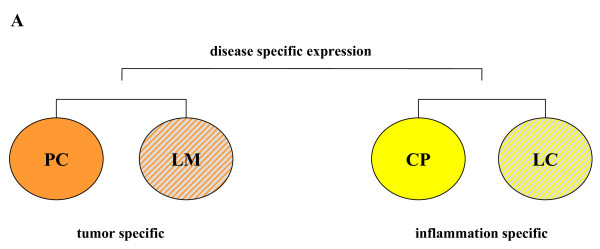
**Schematic expression of the hypothesis: Disease specific expression profile (tumor vs. inflammation) of stellate cells**.

**Figure 3:**
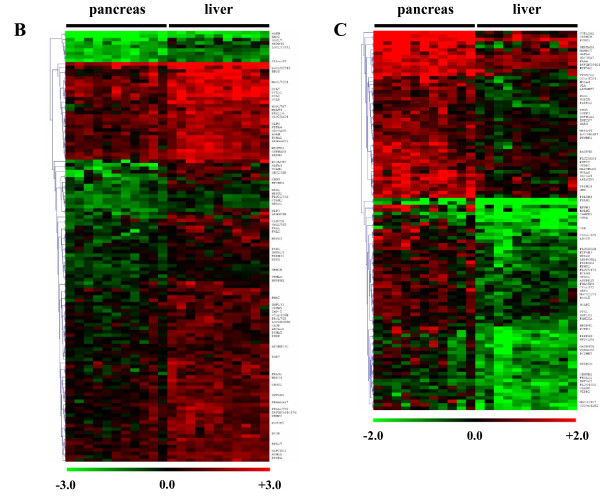
**Heatmaps showing organ specific stellate cell genes: The mRNA expression profile of stellate cells from 22 patients was analyzed to identify organ specific changes**. Heatmaps depict the hierarchically clustered genes that are downregulated in pancreatic stellate cells in comparison to hepatic stellate cells (B) and that are upregulated in pancreatic stellate cells in comparison to hepatic stellate cells (C). Upregulated levels of gene expression are displayed as red bars; downregulated levels are displayed as green bars. Black bars indicate intermediate level of gene expression.

**Figure 4:**
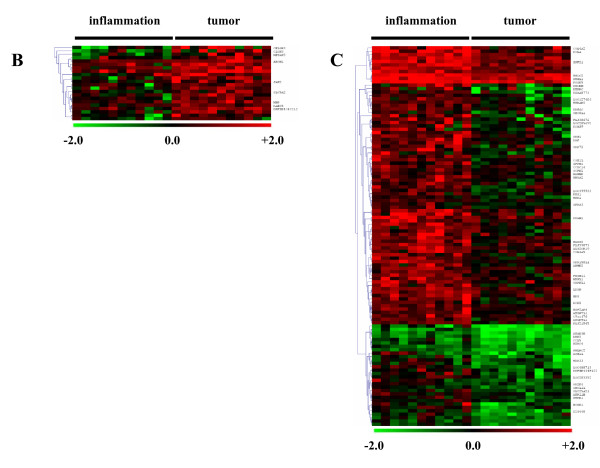
**Heatmaps showing disease specific stellate cell genes: The mRNA expression profile of stellate cells from 22 patients was analyzed to identify disease specific changes**. Heatmaps depict the hierarchically clustered genes that are downregulated in inflamed tissue in comparison to tumor tissue (B) and that are upregulated in inflamed tissue in comparison to tumor tissue (C). Upregulated levels of gene expression are displayed as red bars; downregulated levels are displayed as green bars. Black bars indicate intermediate level of gene expression.

Morphology and purity of stellate cells was routinely checked by immunocytochemistry (Additional file1). In order to identify organ and disease specific transcriptome signatures of stellate cells, a microarray analysis was made (Figure 1, Figure 2). The obtained gene expression profiles were visualized as heatmaps (Figure 3, Figure 4). Red signifies high gene expression, while black signifies intermediate gene expression and green signifies low gene expression of the corresponding gene compared to the reference, respectively. Each row represents one gene, each column represents one sample. The dendrograms on the left side of the heat maps define how the genes cluster after applying hierarchical cluster analysis (average linkage). Expressions of genes are expressed using log2 ratios, where the +/- sign indicates which channel had the higher intensity. Hence, an expression ratio of +2 represents a 4-fold up-regulation of a gene under condition A compared to this gene under condition B, an expression ratio of -2 represents a 4-fold down-regulation.

### Organ specific profile

A total of 160 annotated genes were identified as differentially expressed between pancreas and liver derived stellate cells. To obtain a clear and well-defined matrix, these genes were compared as: downregulated in pancreatic stellate cells in comparison to hepatic stellate cells (n = 80, Figure 3B) or upregulated in pancreatic stellate cells in comparison to hepatic stellate cells (n = 80, Figure 3C). A group of selected genes are presented in Table 2. Significantly different genes in each group with high differential expression ratios were further analyzed by quantitative real time PCR, immunoblotting, immunocytochemistry and immunohistochemistry in all patients.

**Table 2:** A group of highly differentially expressed genes between liver and pancreas stellate cells are shown.

Gene Name Description	RefSeq ID	Fold-change
**ORGAN-SPECIFIC DIFFERENCES IN GENE EXPRESSION**		

**Liver specific transcripts (High in HSC compared to PSC)**		

VCAM1 vascular cell adhesion molecule 1	NM_001078	5.05

PCOLCE2 procollagen C-endopeptidase enhancer 2	NM_013363	4.69

SOX17 SRY (sex determining region Y)-box 17	NM_022454	3.54

GATA4 GATA binding protein 4	NM_002052	3.41

TTC8 tetratricopeptide repeat domain 8	NM_144596	3.02

CCL2 chemokine (C-C motif) ligand 2	NM_002982	2.96

SEC23IP SEC23 interacting protein	NM_007190	2.92

CCL11 chemokine (C-C motif) ligand 11	NM_002986	2.85

**Pancreas specific transcripts (High in PSC compared to HSC)**		

COL11A1 collagen, type XI, alpha 1	NM_080629	13.74

EFNB3 ephrin-B3	NM_001406	4.83

SERTAD4 SERTA domain containing 4	NM_019605	4.29

EGLN2 egl nine homolog 2 (C. elegans)	NM_053046	4.00

CDKN2B cyclin-dependent kinase inhibitor 2B (p15, inhibits CDK4)	NM_004936	3.56

CPE carboxypeptidase E	NM_001873	3.54

CHKA choline kinase alpha	NM_001277	3.31

FBLN1 fibulin 1	NM_001996	3.26

RefSeq ID: Reference Sequence identity; The National Center for Biotechnology Information (NCBI); http://www.ncbi.nlm.nih.gov/RefSeq. The fold-changes display the regulation of expression of the particular gene in stellate cells that is upregulated with respect to comparator.

### Pancreatic stellate cell specific genes

**Figure 5:**
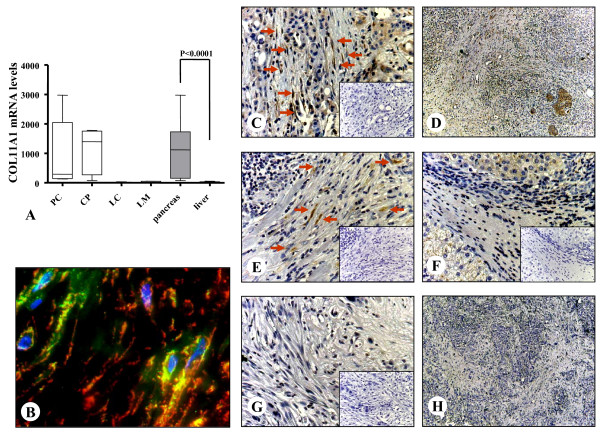
**Quantitative mRNA analysis of Collagen11a1 and its localization in tissues and cultured stellate cells**. Quantitative real-time PCR (A) was performed with the Light Cycler Fast Start DNA SYBR Green kit. The organ specific expression of Collagen11a1 is depicted in the last two columns of the graph. Double-staining and ex vivo immunofluorescence analysis is used to evaluate the colocalization (orange) of the typical stellate cell marker α-SMA (red) and Collagen11a1 (green) in activated stellate cells of pancreatic cancer tissues (B, 630×). Tissues of primary pancreatic ductal adenocarcinoma (C, 200×, D, 50×), chronic pancreatitis (E, 200×), liver cirrhosis (F, 200×) and liver metastasis of pancreatic ductal adenocarcinoma (G, 200×, H, 50×) were immunostained with a specific antibody against Collagen11a1. Negative controls are shown as insets. Arrows show the stellate cells/myofibroblasts in the fibrotic parts of the tissues.

**Figure 6:**
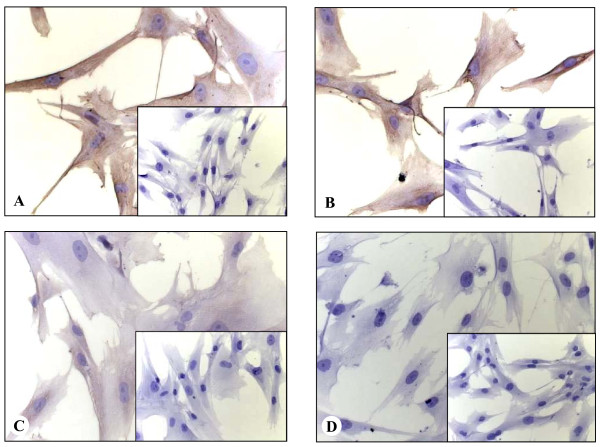
**Expression analysis of Collagen11a1 in cultivated stellate cells: Immunocytochemistry was used to analyze Collagen11a1 expression in cultured stellate cells derived from tissues of primary pancreatic ductal adenocarcinoma (A, 400×), chronic pancreatitis (B, 400×), liver cirrhosis (C, 400×) and liver metastasis of pancreatic ductal adenocarcinoma (D, 400×)**. Cells were seeded on Teflon covered slides, fixed, permeabilized and immunostained with a specific antibody against collagen type 11a1. Negative controls are shown as insets.

In this group, collagen type XI alpha 1 (Col11a1) was the most specific gene with a 13.74-fold upregulation in PSC compared to HSC. In concordance with the array data, Col11a1 was highly pancreas specific with its average mRNA expression being 65-fold (p < 0.0001) higher in the PSC compared to that of HSC as determined by qRT-PCR (Figure 5A). Since there was no suitable antibody for immunoblot analysis, the expression of Col11a1 in tissues and in cultured stellate cells was evaluated by immunohistochemistry and immunocytochemistry. In all patients, PSC showed a specific staining while HSC remained Col11a1 negative by immunohistochemistry. Co-localization of alpha-smooth muscle actin (red) and Col11a1 (green) in stellate cells in pancreatic tissues is shown by immunofluorescence analysis (Figure 5B). There was also a weak staining in pancreatic acini and hepatocytes (Figure 5C-H). Verification of Col11a1 protein expression in cultured stellate cells by immunocytochemistry showed also a PSC specific staining (Figure 6).

### Hepatic stellate cell specific genes

**Figure 7:**
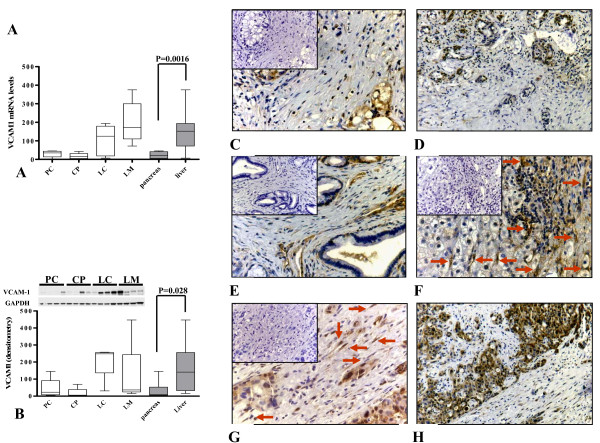
**Quantitative mRNA and protein expression analysis of VCAM1 and its localization in tissues**. Quantitative real-time PCR (A) was performed with the Light Cycler Fast Start DNA SYBR Green kit. Densitometric analysis of immunoblots (B) were performed using the ImageJ program provided by the National Institutes of Health. Optic densities were corrected for the individual background noise and the matching equal loading densities. Each column represents one patient. For each entity, stellate cells from four different patients were evaluated. The organ specific expression of VCAM1 is depicted in the last two columns of each graph. Tissues of primary pancreatic ductal adenocarcinoma (C, 200×, D,50×), chronic pancreatitis (E, 200×), liver cirrhosis (F, 200×) and liver metastasis of pancreatic ductal adenocarcinoma (G, 200×, H, 50×) were immunostained with a specific antibody against VCAM1. Arrows show the stellate cells in the fibrotic parts of the tissues. Negative controls are shown as insets.

**Figure 8:**
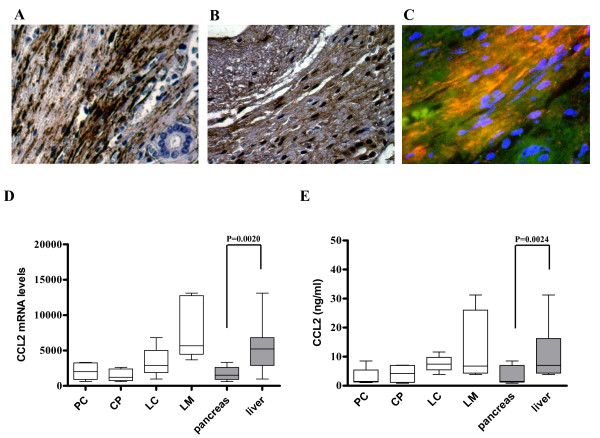
**Co-localization of alpha smooth muscle-actin and VCAM1 molecules in hepatic stellate cells and quantitative mRNA and protein expression analysis of CCL2**. In liver cirrhosis tissues, immunohistochemisty analysis was performed with antibodies against α-SMA (A, 400×) and VCAM1 (B, 400×). Double-staining and ex vivo immunofluorescence analysis was used to evaluate the co-localization (orange) of the typical stellate cell marker α-SMA (red) and VCAM-1 (green) in activated stellate cells of liver cirrhosis tissues (C, 630×). Quantitative real-time PCR (A) was performed with the Light Cycler Fast Start DNA SYBR Green kit. (B) A commercial ELISA kit was used to measure the CCL2 protein in the cell lysates of cultured stellate cells. The disease specific expression of CCL2 is depicted in the last two columns of each graph.

In this group, some genes showed a high HSC specificity. Vascular cell adhesion molecule 1 (VCAM1) was 5.05-fold upregulated in HSC compared to PSC and chemokine (C-C motif) ligand 2 (CCL2) was 2.96-fold upregulated in HSC compared to PSC. In line with the microarray data compared to their average expressions in PSC, VCAM1 and CCL2 mRNA expressions were 5.66-fold (p = 0.0016) and 2.28-fold (p = 0.0020) higher in HSC as determined by qRT-PCR, respectively (Figure 7A & Figure 8D). Next, to quantify the protein expression in vitro, cell lysates of cultured human stellate cells (n = 4 per pathology) were analyzed by immunoblotting or ELISA. Protein expression of VCAM1 in cultivated stellate cells mirrored its mRNA expression. Densitometric analysis of samples showed a 4.71-fold (p = 0.028) higher expression in HSC compared to that of PSC (Figure 7B). Since there was no suitable antibody for immunoblot analysis for CCL2, quantification was made by ELISA. Similar to VCAM1 expression, CCL2 also showed a HSC specific expression irrespective of the pathology (3.15-fold, p = 0.024, Figure 8E). In the last step, we verified the localization of these proteins in human tissues. Liver cirrhosis tissues were probed with alpha-smooth muscle actin (Figure 8A) or VCAM-1 (Figure 8B). Co-localization of alpha-smooth muscle actin (red) and VCAM-1 (green) in stellate cells (orange) in hepatic tissues is shown by immunofluorescence analysis (Figure 8C). All patients showed various degrees of VCAM1 expression. Although immunohistochemistry showed specific staining in stellate cells, there was no obvious organ predilection. In addition to stellate cells, pancreatic cancer cells, hepatocytes and some inflammatory cells were also positive for VCAM1 (Figure 7C-H).

### Disease specific profile

Microarray analysis further identified a total of 89 annotated genes as differentially expressed between stellate cells derived from inflammatory and malignant conditions (Figure 2). To obtain a clear and well-defined matrix, these genes were sorted by two given expression profiles as: downregulated in stellate cells of inflamed tissues compared to stellate cells of tumor tissues (n = 25, Figure 4B) or upregulated in inflamed tissue compared to tumor tissues (n = 64, Figure 4C). Significantly different genes in each group with high differential expression ratios were further analyzed by quantitative real time PCR, immunoblotting, immunocytochemistry and immunohistochemistry in all patients. A group of selected genes are presented in Table 3.

**Table 3:** A group of highly differentially expressed genes between inflammation and cancer-associated stellate cells are shown.

Gene Name Description	RefSeq ID	Fold-change
**DISEASE-SPECIFIC DIFFERENCES IN GENE EXPRESSION**		

**Tumor specific transcripts (High in tumor compared to inflammation)**		

CELSR3 cadherin, EGF LAG seven-pass G-type receptor 3	NM_001407	3.04

SYT13 synaptotagmin XIII	NM_020826	3.04

CLCN3 chloride channel 3	NM_001829	2.69

RTN4RL1 reticulon 4 receptor-like 1	NM_178568	2.45

METAP2 methionyl aminopeptidase 2	NM_006838	2.11

PARD3 par-3 partitioning defective 3 homolog (C. elegans)	NM_019619	2.00

SYK spleen tyrosine kinase	NM_001135052	1.94

JAK2 Janus kinase 2 (a protein tyrosine kinase)	NM_004972	1.93

**Inflammation specific transcripts (High in inflammation compared to tumor)**		

PTPRC protein tyrosine phosphatase, receptor type, C	NM_001846	2.61

DHODH dihydroorotate dehydrogenase	NM_001361	2.56

KIAA0773 KIAA0773 gene product	NM_001031690	2.38

COL4A2 collagen, type IV, alpha 2	NM_001846	2.31

PBX1 pre-B-cell leukemia transcription factor 1	NM_002585	1.70

RBP2 retinol binding protein 2, cellular	NM_004164	1.66

HPX hemopexin	NM_000613	1.64

CCL5 chemokine (C-C motif) ligand 5	NM_002985	1.63

RefSeq ID: Reference Sequence identity; The National Center for Biotechnology Information (NCBI); http://www.ncbi.nlm.nih.gov/RefSeq. The fold-changes display the regulation of expression of the particular gene in stellate cells that is upregulated with respect to comparator.

### Tumor specific genes

**Figure 9:**
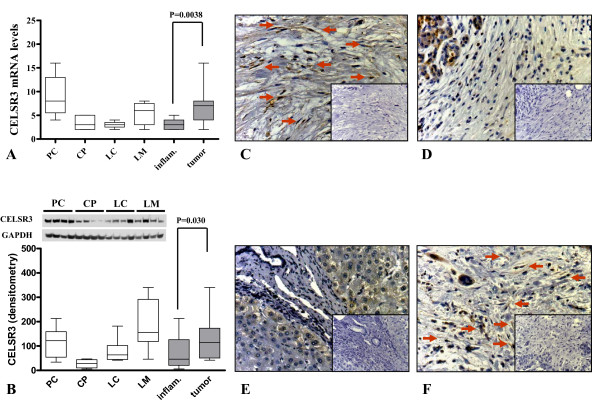
**Quantitative mRNA and protein expression analysis of CELSR3 and its localization in tissues**. Quantitative real-time PCR (A) was performed with the Light Cycler Fast Start DNA SYBR Green kit. Densitometric analysis of immunoblots (B) were performed using the ImageJ program provided by the National Institutes of Health. Optic densities were corrected for the individual background noise and the matching equal loading densities. Each column represents one patient. For each entity, stellate cells from four different patients were evaluated. The disease specific expression of CELSR3 is depicted in the last two columns of each graph. Tissues of primary pancreatic ductal adenocarcinoma (C, 200×), chronic pancreatitis (D, 200×), liver cirrhosis (E, 200×) and liver metastasis of pancreatic ductal adenocarcinoma (F, 200×) were immunostained with a specific antibody against CELSR3. Negative controls are shown as insets. Arrows show the stellate cells in the fibrotic parts of the tissues.

Microarray analysis showed that some genes displayed a cancer specific pattern irrespective of the organ the stellate cells were derived from. For example, cadherin EGF LAG seven-pass G-type receptor 3 (CELSR3) was 3.04-fold upregulated in tumor associated stellate cells compared to inflammation associated stellate cells. Similarly, its mRNA expression was 123% higher (p = 0.004) in the cancer associated stellate cells as determined by qRT-PCR (Figure 9A). By immunoblot analysis, CELSR3 protein was expressed at 83% higher levels (p = 0.030) in tumor related stellate cells compared to that of inflammation related stellate cells (Figure 9B). There was also a disease specific expression of CELSR3 expression in tissues (Figure 9C-F). While hepatocytes were mostly unstained some pancreatic acini and pancreatic cancer cells were also positive for CELSR3.

### Inflammation specific genes

**Figure 10:**
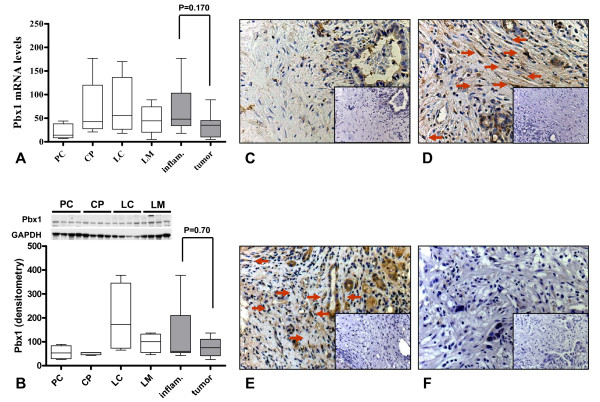
**Quantitative mRNA and protein expression analysis of Pbx1 and its localization in tissues**. Quantitative real-time PCR (A) was performed with the Light Cycler Fast Start DNA SYBR Green kit. Densitometric analysis of immunoblots (B) were performed using the ImageJ program provided by the National Institutes of Health. Optic densities were corrected for the individual background noise and the matching equal loading densities. Each column represents one patient. For each entity, stellate cells from four different patients were evaluated. The disease specific expression of Pbx1 is depicted in the last two columns of each graph. Tissues of primary pancreatic ductal adenocarcinoma (C, 200×), chronic pancreatitis (D, 200×), liver cirrhosis (E, 200×) and liver metastasis of pancreatic ductal adenocarcinoma (F, 200×) were immunostained with a specific antibody against Pbx1. Negative controls are shown as insets. Arrows show the stellate cells in the fibrotic parts of the tissues.

In the microarray analysis, pre-B-cell leukemia transcription factor 1 (Pbx1) was 1.7-fold upregulated in inflammation associated stellate cells compared to tumor associated stellate cells. Although the differences did not reach statistical significance, Pbx1 expression was also 98% higher (p = 0.107) in inflammation associated stellate cells as determined by qRT-PCR (Figure 10A). Similarly, the protein expression of Pbx1 was also 64% higher in stellate cells derived from inflammatory pathologies compared to that of tumor derived stellate cells (p = 0.70, Figure 10B). Although partly discrepant with the immunoblot analysis, this tendency was also visible by immunohistochemistry analysis (Figure 10C-F). In addition to stellate cells, tubular complexes in pancreatic tissues and bile ducts in the liver parenchyma also displayed some Pbx1 positivity.

## Discussion

Here we report the identification of novel tumor stellate cell specific genes and proteins. In addition, hepatic vs. pancreatic stellate cell specific transcripts were discovered. The mRNA and protein expression levels of candidate genes identified by genome-wide transcriptional analysis were confirmed by qRT-PCR, ELISA and Immunoblot analyses. The specific expression pattern of the candidate proteins was further assured *in-vitro *by immunocytochemistry of isolated stellate cells and *ex-vivo *by immunohistochemistry of formalin fixed paraffin embedded tissues. The identified molecular fingerprint of stellate cells might be instrumental in development of novel biomarkers and rational design of therapeutic strategies aiming to selectively target cancer or inflammation associated stellate cells. One drawback of this study is the absence of a third comparator, namely stellate cells from the normal pancreas and liver. However, stellate cells from the normal pancreas do not grow efficiently when propagated by the outgrowth method. Neither could stellate cells from fibrotic tissues like chronic pancreatitis and pancreatic cancer be efficiently propagated by collagenase digestion and centrifugation. The yield of the latter method is very low in comparison to the outgrowth method. Therefore the authors have decided to use only one stellate cell propagation method (outgrowth) to prevent bias that may result from differences in methodology. As a trade-off, a comparator (normal stellate cells) could not be used.

Among the here identified tumor stellate cell specific genes, *JAK2 *and *CELSR3 *pose interesting targets for developing therapeutic strategies. Aberrant JAK2 signaling has been linked to myeloproliferative disorders such as polycythemia vera and chronic myelogenous leukemia [31,32]. Small molecular inhibitors of *JAK2 *signaling have already entered clinical trials. Therefore the role of *JAK2 *overexpression in PSC of PDAC tumors remains to be functionally elucidated. Upregulation of CELSR3 in tumor PSC could also provide a potential druggable target since the protein encoded by this gene is located at the plasma membrane and has intriguing signaling capabilities [33]. CELSR3 is a member of the flamingo protein subfamily which is part of the cadherin superfamily. The flamingo cadherins have nine cadherin domains, seven epidermal growth factor-like repeats and two laminin A G-type repeats in their ectodomain [34]. It is postulated that these proteins are receptors involved in contact-mediated communication, with cadherin domains acting as homophilic binding regions and the EGF-like domains involved in cell adhesion and receptor-ligand interactions. Together, these data suggest an important role for CELSR3 in tumor stellate cells that warrants further investigation.

Recent studies have reported differential regulation of genes in murine and human stellate cells throughout their activation process and under certain drug therapies. Although these studies provide valuable information, a careful interpretation of their data is warranted. First, murine stellate cells may not reflect the situation in humans due to interspecies differences. Second, stellate cells show a great variance depending on the donor, therefore a weakness of the past studies might be the limited number of patients' stellate cells investigated to cover the inter-individual heterogeneity. Our report constitute one of the most comprehensive studies on stellate cell transcriptome using 22 different human donors, thus diminishing potential patient specific biases. This might be a plausible explanation for the success of our approach to identify organ- and disease-specific stellate cell transcriptome. In contrast to the studies comparing quiescent vs. activated stellate cells, we compared human stellate cells that were activated *in-vivo *either by chronic inflammation or cancer. Currently there is no information on how the "ancestral microenvironmental activity" of stellate cells affects their transcriptome after *in-vitro *cultivation. Therefore, the identified tumor stellate cell specific genes e.g. CELSR3 might provide a favorable therapeutic profile to selectively target tumor stroma while sparing the stellate cell activity under physiological conditions.

It has been reported that experimental ablation of tumor associated fibroblasts or down-regulation of the hedgehog-signalling pathway in the pancreatic tumor stroma decreased cancer cell growth and greatly increased intra-tumoral uptake of chemotherapeutic drugs in murine models of colon, breast cancer an pancreatic cancers [5,35]. It is likely that targeting the stroma in order to uncouple stromal-cancer cell interactions may interrupt multiple aberrant autocrine and paracrine pathways that promote pancreatic cancer cell growth, invasion and metastasis [4,21]. Currently, there is no stellate cell specific promoter known to specifically target the stromal cells in the liver or pancreas. Moreover, the similarities between PSC and HSC make it very difficult to target one population without creating side-effects on the other one. For example, the usage of retinoids seems promising in deactivating PSC *in-vitro *[36]. In contrast, treatment with retinoids were shown to provoke fibrogenic effects in HSC, thus, limiting its efficient systemic use in the clinical setting [37].

In this context, our data provide valuable information focusing on the subtle but important differences of liver vs. pancreatic stellate cells that exist rather than their similarities. We found collagen type 11a1 expression to be highly specific for the PSC and almost absent in HSC. Type XI collagen is a minor fibril-forming collagen that assists in proper type II collagen fibril formation [38]. Col11a1 is mainly expressed in articular cartilage and the vitreous fluid of the eye [39-43]. During embryogenesis, Col11a1 expression is also detectable in many other human fetal tissues including the bone [40]. Marshall and type II Stickler syndromes are genetically transmitted diseases that are caused by mutations in Col11a1 gene manifested with sensory and skeletal abnormalities [44,45]. In Col11a1 knockout mice, chondrocytes fail to fully differentiate causing a chondrodystrophic phenotype with skeletal abnormalities. These data show that Col11a1 is essential for skeletal morphogenesis because it controls type II collagen fibrillogenesis, chondrocyte maturation and bone mineralization [46,47]. Importantly, in adult life these tissues are not undergoing dynamic changes. Therefore Coll11a1 may provide an interesting target for conditional knockout to target PSC in order to assess PSC contribution in conditions like chronic pancreatitis or pancreatic cancer.

Buchholz et al. compared gene expression of human HSC, PSC and skin fibroblasts [9]. Their data show that, compared to dermal fibroblasts, HSC and PSC exhibit great similarities in their transcriptional phenotypes and possibly share a common origin [3,9]. Indeed, HSC and PSC are activated by common cytokines, growth factors and alcohol metabolites during development of tissue fibrosis [3,7,30,48-50]. However, the fundamental differences in their microenvironments may condition these cells to differentially respond to organ injury and cancer [1,3,30]. For example, in contrast to hepatocellular carcinoma, desmoplasia is a typical feature of pancreatic ductal adenocarcinoma. This difference may result in part due to the release of potent fibrogenic mediators by pancreatic cancer cells [1,19,30,51]. We have previously shown that, compared to the primary tumor, liver metastasis of PDAC elicit a weaker fibrogenic response in the HSC that surround them. This observation can be due to the differences of HSC and PSC, or it could, considering the temporal sequence of events, merely reflect a shorter cancer-HSC interaction [30]. Interestingly, we found almost twice as much differentially regulated genes with an organ specific expression pattern as compared to chronic inflammation or tumor related genes. Moreover, differences in gene expression levels between HSC and PCS were more pronounced compared to disease specific stellate cells. These differences can be due to various factors. It may reflect that PSC and HSC do not share a common origin, or it may also suggest that chronic inflammation and cancer develop in similar microenvironments created by the activated stroma of the respective organs.

In the clinical setting, chronic inflammatory conditions of the liver (cirrhosis) and pancreas (chronic pancreatitis) not only increase the incidence of cancer, but also constitute diagnostic hurdles. Biopsies that do not show tumor structures are not helpful in diagnosis since they are useless in exclusion of the tumor. However, identification of tumor versus inflammation specific signatures of the stroma may increase diagnostic accuracy of biopsies even where tumor tissue is missed.

Conclusively, identification of inherent and acquired differences in stellate cell transcriptome will improve current understanding of stellate cell pathophysiology and may facilitate the discovery of novel selective therapeutic targets. Our data provide a new platform to understand and possibly interfere with organ or disease specific responses of stellate cells. Further research is warranted to analyze the importance of these findings at functional levels.

## Abbreviations

CP: Chronic pancreatitis; HSC: Hepatic stellate cells; IHC: Immunohistochemistry; LC: Liver cirrhosis; LM: Liver metastasis; PDAC: Pancreatic ductal adenocarcinoma; PSC: Pancreatic stellate cells.

## Conflict of interests

The authors declare that they have no competing interests.

## Authors' contributions

Study concept and design: ME, HF, JD, PEH, AA, JK; acquisition of data: NW, TS, ZP, CS, XJ, UW, NAG, WA; analysis and interpretation of data: ME, CS, WA, AA, JK; drafting of the manuscript: ME, AA, JK; critical revision of the manuscript for important intellectual content JD, PEH, HF; statistical analysis: CS; obtained funding: NAG, PEH, AA, ME, JK; technical and material support: NAG, JD; study supervision: AA, JK. All authors have read and approved the final manuscript.

## Supplementary Material

Additional file 1 **Expression profile of stellate cells for typical markers.** The purity of the stellate cell population was routinely checked by immunocytochemistry and immunofluorescence analyses at every passage used. Cells were seeded on Teflon covered slides, fixed, permeabilized and immunostained with specific antibodies against α-SMA, collagen type-Ia, fibronectin, periostin, collagen XVIII and VEGF as published before [30]. Contamination profiling for cancer cells was made by a specific antibody against Pan-cytokeratin. Non-immunized IgG was used appropriately as negative control (original magnification: 200-400×). Click here for file
