# Supplementary material for: Organ-, inflammation- and cancer specific transcriptional fingerprints of pancreatic and hepatic stellate cells
Source: Mol Cancer. 2010 Apr 23;9:88. doi: 10.1186/1476-4598-9-88 (PMC2876060; doi:10.1186/1476-4598-9-88)
Supplement: Additional file 1 — Expression profile of stellate cells for typical markers. The purity of the stellate cell population was routinely checked by immunocytochemistry and immunofluorescence analyses at every passage used. Cells were seeded on Teflon covered slides, fixed, permeabilized and immunostained with specific antibodies against α-SMA, collagen type-Ia, fibronectin, periostin, collagen XVIII and VEGF as published before [30]. Contamination profiling for cancer cells was made by a specific antibody against Pan-cytokeratin. Non-immunized IgG was used appropriately as negative control (original magnification: 200-400×). [file 1476-4598-9-88-S1.PDF]

Additional file-1

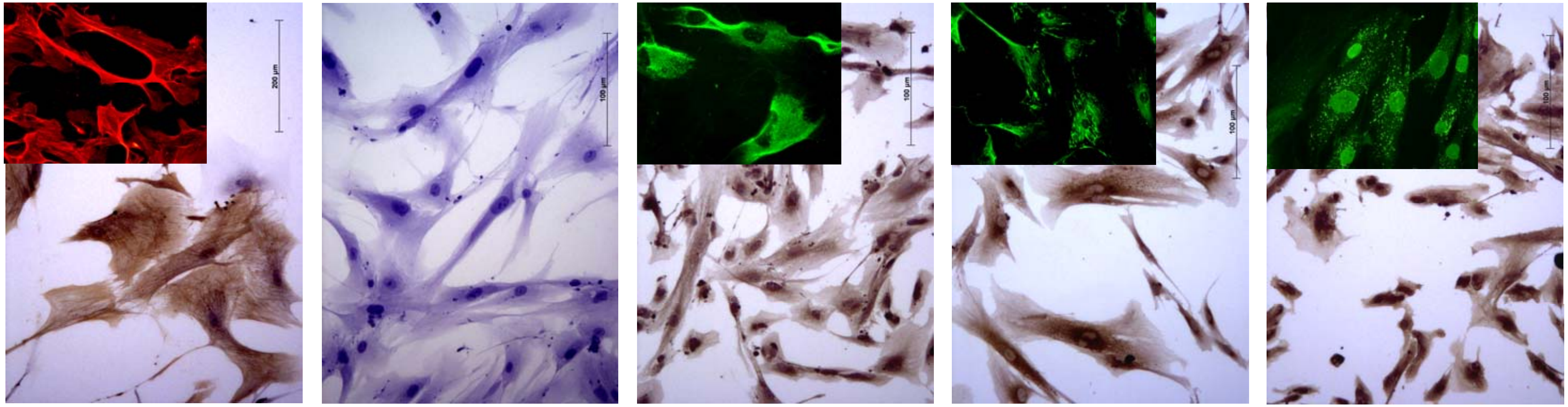

a-SMA | Pan-cytokeratin | Collagen-Type Ia | Fibronectin | Periostin

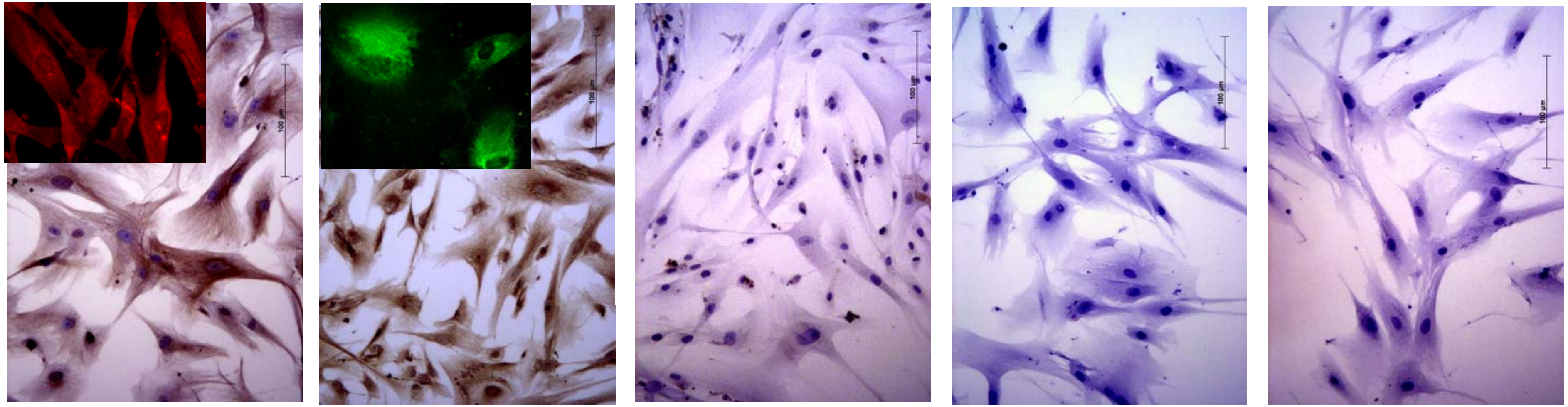

Collagen-XVIII | VEGF | Goat-NC | Mouse-NC | Rabbit-NC
